# A systematic review of the unit costs of allied health and community services used by older people in Australia

**DOI:** 10.1186/1472-6963-13-69

**Published:** 2013-02-20

**Authors:** Inez Farag, Cathie Sherrington, Manuela Ferreira, Kirsten Howard

**Affiliations:** 1 George Institute for Global Health, University of Sydney, 321 Kent Street, Sydney, NSW 2006, Australia; 2 Sydney School of Public Health, University of Sydney, Sydney, NSW, 2006, Australia

**Keywords:** Unit costs, Allied health services, Community services

## Abstract

**Background:**

An economic evaluation of interventions for older people requires accurate assessment of costing and consideration of both acute and long-term services. Accurate information on the unit cost of allied health and community services is not readily available in Australia however. This systematic review therefore aims to synthesise information available in the literature on the unit costs of allied health and community services that may be utilised by an older person living in Australia.

**Method:**

A comprehensive search of Medline, Embase, CINAHL, Google Scholar and Google was undertaken. Specialised economic databases were also reviewed. In addition Australian Government Department websites were inspected. The search identified the cost of specified allied health services including: physiotherapy, occupational therapy, dietetics, podiatry, counselling and home nursing. The range of community services included: personal care, meals on wheels, transport costs and domestic services. Where the information was not available, direct contact with service providers was made.

**Results:**

The number of eligible studies included in the qualitative synthesis was fourty-nine. Calculated hourly rates for Australian allied health services were adjusted to be in equivalent currency and were as follows as follows: physiotherapy $157.75, occupational therapy $150.77, dietetics $163.11, psychological services $165.77, community nursing $105.76 and podiatry $129.72.

**Conclusions:**

Utilisation of the Medicare Benefits Scheduled fee as a broad indicator of the costs of services, may lead to underestimation of the real costs of services and therefore to inaccuracies in economic evaluation.

## Background

A fundamental requirement for economic evaluation is accurate assessment of costing [1,2]. This allows comparative analysis of the impact of an intervention program on the costs of service utilisation and ultimately the accrued cost to benefit ratio [2]. Information on the costs of hospital and medical services is readily available [3]. For example, the costs of injuries that are associated with the most severe mortality, morbidity and hospitalisation have been established using data from hospital databases on cost of hospital admission, cost of ambulance transport, emergency department and medical service utilisation.

An economic evaluation requires more than consideration of the acute or short term provision of services however [4]. The more widespread and longer term consequences of illness, injury and disability, with the follow-on effect of health service use, have to be considered. The difficulty in the use of available data however is that reliance on established fee schedules, for example, the Medicare Benefits Schedule in Australia [5] may not provide an accurate indication of the real cost of providing services in some clinical areas [6]; this may result in serious underestimation of the actual costs of service delivery [7]. In addition for many community services there is no established fee structure and as a result there is great variability in the unit costs that may potentially be used in economic evaluation. To fully appreciate the cost of age-related disease and the cost effectiveness of healthcare programs, information on the cost of the full range of services utilised (including allied health and community services) is required.

The primary purpose of this systematic review, which is the first study of its type in Australia, is to provide a consolidated resource of costs of allied health and community services used by older people in Australia. This will facilitate the inclusion of these costs in future economic evaluations.

## Method

### Literature search

To identify the unit costs of services a comprehensive search of the databases Medline, Embase, Cumulative Index to Nursing and Allied Health Literature (CINAHL) Google Scholar and Google was conducted. In addition specialised economic databases were reviewed and accessed through the Centre for Reviews and Dissemination allowing exposure to relevant databases including Database of Abstracts of Reviews and Effects (DARE) and 11,000 economic evaluations collected in the National Health Service Economic Evaluation Database (NHSEED). It was considered that the relevant information may also be contained within government departmental literature and as such a search was conducted of Australian Government Department websites, including: NSW Department of Health, Commonwealth Department of Health and Ageing, Veteran’s Affairs and the National Health and Medical Research Council. Where insufficient information was available a review of Department of Health websites of other Australian States was to be conducted. The NSW Injury Risk Management Research Centre and Australian Institute of Health and Welfare websites were also searched. The search was to identify cost of specified allied health and community services from 1980 to date. An exception to this rule was applied however, for certain databases such as Google Scholar and Google that yielded large results; to ensure feasibility, the search was limited from 2000 to date.

The allied health services that were investigated included: physiotherapy, occupational therapy, dietetics, podiatry, community nursing and counselling. Community services comprised of personal care, meals on wheels, transport services and domestic services including home cleaning, gardening services and general handyman rates. Staff travel was included as it is often a required cost item in economic evaluation. For international data, other allied health services were included, which appeared to be frequently utilised and had available costs reported. Respite care and estimates of family care costs were included as they were considered to be necessary cost items in economic evaluations. Excluded from the systematic review were ambulance, emergency department, time in hospital costs, medical practitioner consultations and residential care facility costs.

Included in the search terms were keywords: unit cost, unit price, schedule of fees, allied health services (range of terms of single professions and combined terms) and community services (range of terms of single services).

In addition the reference lists of retrieved articles were searched.

### Inclusion of studies

Included were peer-reviewed published studies or publicly available reports that included an estimate of the unit cost of services. Cost of injury studies and economic analyses associated with randomised controlled trials were included if the content included information on the unit costs of services. Whilst the predominant interest is in the range of services utilised by the “older” population, papers and reports were not excluded if the relevant information on the unit costs related to the provision of similar services to a younger population.

Although the focus of the study was on Australian costs, overseas estimates were also summarised, to complement the Australian data. International costs were converted to 2011 Australian dollars using Organisation for Economic Co-operation and Development (OECD) purchasing power parities [8].

### Quality of included studies

There was no limitation made or elimination of studies based on study quality as this factor did not impact on the accuracy of the unit costs utilised; often the information on unit costs was contained in the Appendix of relevant papers.

### Contact with service providers

Direct contact with providers of domestic, gardening and handyman services in Australia was made to obtain the information on costs that was lacking in the literature. On average ten service providers were contacted from each category (full details of the providers contacted is available on request).

### Data extraction

A standardised form was used to extract information from the published manuscripts included in the systematic review (available on request). Extracted information included: study design, cost of range of allied health and community services, source of cost item information, currency and the year of cost collection. Costs that were not relevant to the study were not incorporated. Screening of abstracts and titles and subsequent full text evaluation was conducted by IF; a sample of the manuscripts (10%) were examined by two reviewers (IF, MF). Any disagreements were resolved by consensus; where consensus was not reached, input from a third examiner was sought (CS).

### Data synthesis

The studies reported costs in different currencies and different years; this information was standardised by conversion to Australian dollars for the 2011 cost year. The process included conversion to Australian dollars, where necessary, using purchasing power parities [8] and inflation of the values to 2011 prices using the “health price index” [9].

Studies varied in the method of reporting unit costs, some opting to describe the cost per occasion of service [10]; whilst others describe an estimated hourly rate and others still a cost value per minute [11]. For some disciplines, such as physiotherapy and dietetics, an estimated duration was allocated to the occasion of service in some studies [6,12]. For other disciplines, such as psychological counselling services, sessions were described in terms of “simple” or “complex” interventions [13,14].

The unit costs of different services from the various studies and government reports were extracted. In addition a conversion to a cost per hour was made, where possible, using the estimated duration of the occasion of service provided. Where a publication provided a cost per occasion of service, this was multiplied by 2.5 (session ranging in time between 20–30 minutes), providing an average of the hourly rate charged.

Overall the methodology and reporting of this study adhere to the PRISMA guidelines for conducting qualitative systematic reviews [15].

## Results

**Figure 1:**
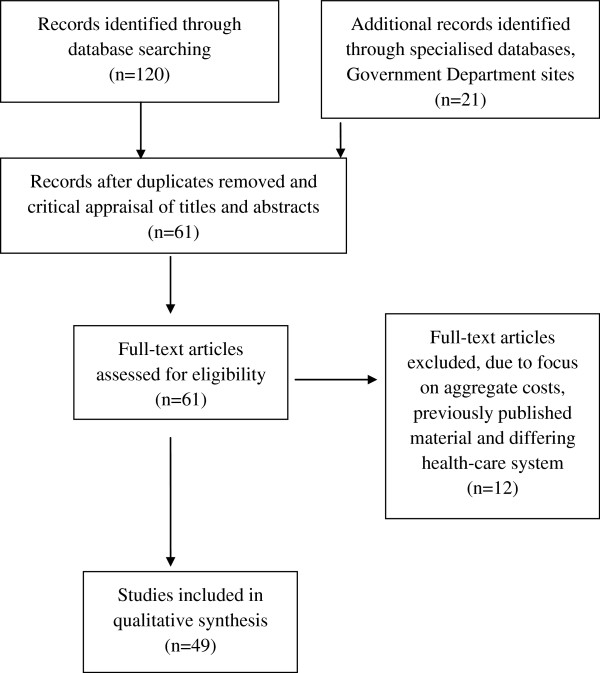
Flow chart of the search and retrieval process.

The flow chart of the search and retrieval process is displayed in Figure 1. One hundred and twenty articles were identified as being potentially relevant from the general databases and 21 from the combination of other sources. Following elimination of duplicated articles and the appraisal of titles and abstracts, 61 full text articles were identified as potentially relevant. In the final qualitative analysis twelve full-text articles were eliminated as there was a focus on aggregated rather than unit costs (five studies) and seven studies relied on unit costs from previously utilised published material. A review of the full-texts identified 49 eligible studies or reports; 28 relevant articles were derived from the general databases, sixteen papers were identified from the economic-specific databases and two publications were identified as relevant from Australian government department websites. A review of the reference lists of obtained articles yielded three studies of relevance.

### Study characteristics

Of the eligible studies 15 were Australian based, 18 were based in the United Kingdom, 9 based in other countries of the European Union, 3 based in the United States, 1 based in Canada, 1 based in Hong Kong and 2 were based in New Zealand.

Of the 15 Australian based studies/reports, seven (47%) were published prior to the year 2000, the first study in 1989 [16] (published in 1996). Many of the Australian studies could be described as descriptive reviews and case studies (n=5), the remainder having the following study design: cost of injury studies (n=3), resource utilisation studies (n=2), economic evaluations (n=2), decision analysis modelling (n=1) and Government Department Schedule of Fees (n=2). Equal number of studies described the unit costs on an hourly basis as per session/visit basis (n=5); whereas at least four distinguished between the cost of an initial consultation and subsequent visits [3,6,10,17]. Several studies included information from local service providers and were specific to a particular environmental context [18,19]; this affects the ability to generalise the information to an alternate setting. Others derived costs from surveys of private practitioners [6,17,20] and here the validity of the cost estimates was influenced by the size of the study.

Most of the economic evaluations retrieved from the specialised databases (n=16) were conducted in the United Kingdom (n=11, 69%); these varied in relation to the comprehensiveness of the economic evaluations conducted. Several studies tended to rely on unit costs on the established work of Curtis & Netten [21].

### Unit costs of services in Australia

Table 1 contains the unit costs of a range of allied health and community services in Australia. The calculated hourly rates for allied health services were within the range $105.67 (community nursing) to $165.77 (psychological counselling services). The results suggest considerable variety in the costs charged by private practitioners of allied health services in Australia. For all practitioners a higher fee is charged for the first consultation, than for subsequent visits. The costs charged are closely associated with the nature of the service provided and whether it is, for example, provided in the home environment or as part of a group intervention.

**Table 1:** Unit costs of allied health and community services in Australia

**Services**	**Cost given by individual studies 2011 $AD**	**Mean (SD), range**
**Physiotherapy**		
Public Hospital Service	$50.10 [18], $76.10 [20] $73.40 [10], $72.14 [22]	$67.94 per hour (12.00) $41.26-$76.10
Group	$29.09 [23], $27.35 [24]	$28.22 per patient (1.23)
Hydrotherapy	$15.77 [20], $35.80 [24]	$25.79 per patient (14.16)
Outreach	$212.19 [18]	$212.19
Private per visit	$76.50 [19], $51.44 [20]	$ 63.97per consultation (17.72)
First consultation	$96.32 [23], $100.40 [10], $91.18 [17], $69.24 [3]	$89.29 per consultation (13.89) $69.24-$100.40
Second consultation	$61.63 [10], $70.14 [17], $55.39 [3]	$62.39 per consultation (7.4) $51.44-$70.14
Medicare Benefits Schedule	$59.90-Fee $50.95- Rebate [5]	$55.43 per consultation
Special Population Veterans’ Affairs	$60.53 [17], $61.10 (initial & standard) [24]^1^	$60.82 per consultation ($60.53-$76.70)
**Hourly rate**		**$157.75 per hour**
Percentage difference between the private and the MBS fee		62-89% of the private fee
Fee suggested by professional association (National)		$79.25 (initial consultation) $66.95 (subsequent visits)
**Occupational Therapy**		
Private per visit	$71.30 [23], $76.50 [19], $76.10 [20], $37.81 [25], $69.24 [3] (initial), $55.39 [3] (subsequent)	$64.39 per consultation (15.12) $37.81-$85.80
Medicare Benefits Schedule	$59.90-Fee $50.95- Rebate [5]^2^	$55.43 per consultation
Special Population Veterans’ Affairs	$61.10 (initial & standard) ^3^	$61.10 per consultation $61.10-$111.25
Group Therapy	$21.95 per person [24]	$21.95 per person
**Hourly rate**		**$150.77 per hour**
Percentage difference between the private and the MBS fee		86-146% of the private fee
Fee suggested by professional association (Victoria)		$120.00 per consultation (expected 1 hour duration)
**Dietetics**		
Private per visit	$60.89 [6]	$60.89 per consultation
First consultation	$82.28 [7], $105.16 [17]	$93.72 per consultation (16.18)
Second consultation	$61.71 [17]	$61.71 per consultation
Medicare Benefits Schedule	$59.90 - Fee $50.95- Rebate [5]	$55.43 per consultation
Special Population Veterans’ Affairs	$85.30 (initial), $61.10 (standard) [24]^4^	$73.20 per consultation
**Hourly rate**	$182.66 [7]	**$163.11 per hour**
Percentage difference between the private and the MBS fee		53-91% of the private fee
Fee suggested by professional association		Private practitioner survey $70-$130 per hour
**Psychological Services**		
Private per visit	$93.56 [23]	$93.56 per consultation
Medicare Benefits Schedule	$59.90- Fee $50.95- Rebate [5]^5^	$55.43 per consultation
Special Population Veterans’ Affairs	Psychologist	
	$69.35 (20–50 minutes) ^6^	
	$97.90 (50 + minutes)	
	$146.90 (90 + minutes)	
	Clinical Psychology	
	$97.90 (30–50 minutes) ^7^	
	$143.70 (50 + minutes)	
	$215.60 (90 + minutes	
**Hourly rate**		**$165.77**
Percentage difference between the private and the MBS fee		59% of the private fee
Fee suggested by professional association		$222.00 per one hour consultation
**Community Nursing**		
Private per visit	$74.37 [20], $37.81 [26]	$56.09 per consultation (25.85)
Per hour	$69.24 [3], $82.47 [16], $28.85 [22]	$60.19 per hour (27.93)
Special Population Veterans’ Affairs	Average^8^	
	$40.47 ($37.65- $43.28) -20 minute visit	
**Hourly rate**		**$105.76 per hour**
Percentage difference between the private and the MBS fee		80-118% of the private fee
Fee suggested by professional association		No recommended fee by association Private agencies $46.94-$69.40 per hour
**Podiatry**		
Private per visit	$22.95 [20]	$22.95 per consultation
First consultation	$66.85 [17]	$66.85 per consultation
Second consultation	$61.71 [17]	$61.71 per consultation
Medicare Benefits Schedule	$59.90- Fee $50.95- Rebate [5]	$55.43 per consultation
Special Population Veterans’ Affairs	$61.10 (standard) [24]^9^	$61.10 per consultation
**Hourly rate**		**$129.72**
Percentage difference between the private and the MBS fee		79-92% of the private fee
Fee suggested by professional association		Based on market demand ($60-$70 per consultation)
**Staff travel**	0.38c per km [19], 0.73c per km [26], 0.69c per km (0.63c per km- 0.75c per km) ^11^	**0.60c per km (0.17)**
**Community Services**		
**Personal care**	$34.23 [20], $28.07 [26], $46.89 [16] per hour	**$36.40 per hour (9.6)**
**Meals on wheels**	$6.56 per meal [20], $20.00 [16], $6.75 ($4.50-$9.00) ^10^	**$11.10 per meal (7.71)**
**Patient transport**	$6.56 per trip (council/hospital provided) [20], $18.22 per trip [16]	**$12.39 per trip (8.25)**
**Domestic services**	$18.36 per service [20], $38.31 per service [16]	
	Domestic care rates per hour ^12^	
	**$39.07** (Monday-Friday)	
	**$48.52** (Saturday)	
	**$57.31** (Sunday)	
	Gardening **$52.26** ($30.00-$60.00) per service (10 providers)	
	Handyman rates **$50.80** ($40-$80.30) per service (10 providers)	

^1 ^$64.74 extended consult, $65.65 standard in institution, $76.70 extended consult (home).

^2^Specialist OT services (mental health) $50.95-$59.90 (20–50 minutes) $71.95-$84.60 (50 minutes +).

^3^$111.25 (50+ minutes- out of rooms).

^4 ^$106.60 (extended).

^5 ^Specialist Psych services - $57.80-$68.00 (20–50 minutes), $81.60-$96.00 (50 + minutes) Clinical Psychology- $81.60-$96.00 (30–50 minutes), $119.80-$140.90 (50+ minutes).

^6 ^Out of rooms - $94.35 (20–50 minutes).

^7 ^Out of rooms $122.35 (30–50 minutes), $168.15 (50+ minutes), $252.25 (90+ minutes), Neuropsychological Assessment (1–4 hours) $574.80.

^8^ Complex community nursing schedule of fees, which is dependent on the type of service provided, including: clinical mentoring, medication administration, palliative care and personal care.

^9 ^Out of rooms $68.95.

^10 ^Direct contact with service provider – meals on wheels NSW.

^11^Direct contact with Australian Taxation Office.

^12 ^Direct contact with service providers (11 providers).

An average duration per occasion of service has been clarified for the disciplines of physiotherapy and dietetics [5,6,24] and is estimated to be between 20–30 minutes; but is less clear for other disciplines. Session duration appears to have an impact on the service costs [5,24] whether this is expressed in terms of the time taken per session as for physiotherapy or in the complexity of the intervention required, as for counselling [14].

The Medicare Benefits Schedule fee is lower than the private set fee for most disciplines. The percentage discrepancy between the MBS fee and the private fee is greatest for psychological services at 59% and lowest for community nursing 80-118% (Table 1). There is indication however of flexibility in fee structure in the private setting to accommodate special populations, including clientele of the Department of Veterans’ Affairs and those supported by the Medicare Benefits Scheme.

There are other factors that impact on costing. Public hospital services, for example, are generally costed at a lower rate in comparison to private practitioner fees [18,20]. For some disciplines specialist qualifications are compensated at a higher rate by the Medicare Benefits Scheme (MBS) whereas for nursing services, cost is dependent on the complexity of services provided [24]. There has been a steady increase in the cost of service delivery for all allied health services. The percentage increase in physiotherapy being an increase of 4.00% during 2011–2012, in comparison to an increase of 2.6% during 2010-2011 [27]. Most of the professional associations choose not to become involved in fee setting (Dietitians Association of Australia) but do provide members with information on average fees charged by private practitioners of their discipline (Table 1). The Australian Psychological Society produces a schedule of recommended fees for psychological services [28].

Patient transport costs were dependent on subsidy from local or health services [20]. Domestic services, including home cleaning, gardening and handyman services are greatly influenced by the time of service delivery, with higher rates charged for work conducted on Saturday and Sunday.

### International unit costs of services

Many of the studies were conducted in the United Kingdom and most utilised the reference document developed by Netten & Curtis [21], which categorises in considerable detail the unit costs of health services. The results of the unit costs for the international data are contained in Table 2. There is variability in the costs of allied health and community services between countries, including across countries of the European Union. The work of Lafuma [29] highlights the different costs attributed to home care services between, Italy, France, Germany and The United Kingdom. International data included a wider range of reported allied health service costs with information available on the costs of social work and speech pathology. Respite care was a reported cost item in the United Kingdom, whereas estimates of the cost of family care were available for the United Kingdom and the United States. Respite care costs in the United Kingdom are highly variable and dependent on subsidy from health or local services [30].

**Table 2:** Unit costs of allied health and community services in selected countries

**Service**	**Cost given by individual studies 2011 $AD**	**Mean (SD), range**
**United Kingdom**		
Counselling	$77.89 [31], $111.25 [31]^1^	$94.57 per consultation (23.59) $77.89-$111.25
Home care	$19.16 [31], $44.23 [32], $17.75 [33], $24.31 [34], $27.72 [35]	$26.63 per hour (13.04) $17.75- $44.23
Podiatry	$46.97 [32], $28.70 [36], $53.67 [35]	$43.11 per consultation (12.92) $28.70-$53.67
Dietetics	$135.67 [32]	$135.67 per consultation
Home nursing	$46.62 [32], $34.50 [33], $55.89 [37], $112.42 [38], $38.69 [34], $41.28 [39]	$54.90 per consultation (29.13) $34.50-$112.42
Physiotherapy	$24.29 [35], $41.97 [40], $26.58 [39]^2^, $48.13 [41], $64.23 [42]^3^, $35.16 [43], $34.31[12]^4^, $54.00 [44]	(NHS) $41.08 per consultation (13.77) $24.29-$64.23
	$62.48 per hour [21]	(NHS) $62.48 per hour
	$104.14 [42]^5^, $45.40 [34], $34.22 [21]^6^, $45.34 [12], $106.97 [32], $104.27 [41], $83.30 [21]^7^	(Private) $74.81 per consultation (32.19) $34.22-$104.14
	$161.39 [37]	(Private) $161.39 per hour
Occupational Therapy	$106.97 [32], $24.29 [35], $45.32 [34] $547.88 [45] per day,	$58.86 per consultation (42.97) $24.29-$106.97
Social Work	$216.55 [32], $278.61 [35]	$247.58 per consultation (43.88)
Speech Pathology	$43.82 [34]	$43.82 per consultation
	$99.14 [32], $96.40 [35]^8^	$97.77 per hour (1.94)
Meals	$5.87 [35], $9.82 [34]	$7.85 per meal (2.82)
Family care	$26.78 [34]	$26.78 per hour
Respite care	$254.32 [30] (NHS), $63.57 (VS), $117.86 (LCA) [30]^10^	$145.25 per day (98.28) $63.57-$254.32
**Europe**		
Home care	$25.67 [29] (FR), $34.01 [29] (GR), $30.32 [29] (UK), $48.57 [46], $52.64 [47], $55.21 [48]	$41.07 per consultation (12.59) $25.67-$55.21
	$60.15 [49]	$60.15 per hour
Home Nursing	$35.05 [50], $80.20 [49], $137.95 [46], $65.87 [51], $151.64 [47]	$94.14 per consultation (49.27 $35.05-$151.64
Physiotherapy	$85.49 [46]^11^, $76.44 [47] ,$36.75 [52]^12^	$66.23 per consultation (25.93) $36.75 -$85.49
	$50.13 [53], $65.87 [51], $94.47 [48]	$70.16 per hour – public (22.48) $50.13-$94.47
Occupational Therapy	$94.47 [48], $99.09 [46]^13^	$96.78 per consultation (3.27)
	$50.13 [53], $65.87 [51]	$58.00 per hour- public (11.13) $50.13-$65.87
Staff travel	0.38c per km [50], 0.36c per km [53]	0.37c per km (.01) 0.36-0.38c per km
Meals	$14.03 [49]	$14.03 per meal
Cleaning	$54.13 [49]	$54.13 per hour
**United States**		
Counselling	$179.40 [14] per occasion of service	$179.40 per consultation
Home care	$10.36 [54] per hour	$10.36 per hour
Home nursing	$35.00 [54] per hour	$35.00 per hour
Physiotherapy	$32.96 [54] per occasion of service	$32.96 per consultation
Social work	$26.04 [54] per hour	$26.04 per hour
Family care	$8.64 [54], $20.41 [55]	$14.53 per hour (8.32) $8.64-$20.41
**Canada**		
Home care	$26.53 [56]	$26.53 per hour
Home nursing	$67.56 [56]	$67.56 per consultation
Physiotherapy	$111.21 [56]	$111.21 per consultation
Occupational Therapy	$138.32 [56]	$138.32 per consultation
**Hong Kong**		
Home nursing	$65.11 [57]	$65.11 per consultation -public
**New Zealand**		
Home nursing	$20.80 [58]	$20.80 per hour
Physiotherapy	$24.22 [58]	$24.22 per hour
Occupational Therapy	$27.85 [59]	$27.85 per consultation-public
Staff travel	0.78 per km [58], 0.69 per km [59]	0.74c per km (.06) (0.69-0.78)c per km

^1^cost dependent on level of complexity of the case.

^2^Service is 20 minutes- $28.43 for inpatient.

^3^Initial consultation, $32.10 subsequent visits.

^4^ Service is 20 minutes, Home visit $111.27.

^5^Initial consultation, $87.72 subsequent visits.

^6^ 20 minute service.

^7^Clinical specialist.

^8^ $1.94 per minute (speech pathology) and $2.48 per minute (Home nursing).

^9^ Initial consultation, $87.72 subsequent visits.

^10^Cost is dependent on provider of service, NHS, voluntary sector or local council authority.

^11^ $101.03 for home visit.

^12^ Service is 30 minutes in duration.

^13^$101.03 for home visit.

## Discussion

The costs of allied health and community services are a significant component of the total cost of illness, injury and disability for the older person [56] and often these costs are overlooked in economic evaluation. Accurate estimation of the unit costs of services specific to the nature and type of service provided, for example whether it is a group service, first consultation or provided by the public health sector, allows for a more precise calculations of the costs incurred.

The results of this review suggest variability in the cost structure of allied health services in Australia. Whilst the Medicare Benefits Schedule fee is at the lower end of the fee structure with noted discrepancies in the percentage differences between the differing disciplines, there is suggestion of flexibility within the private health sector to cater for special populations [17]. Nevertheless reliance on the fee established by the Medicare Benefits Scheme in estimation of the costs of allied health services in economic evaluation may lead to a serious underestimation of the cost of services, with sometimes as much as double the cost being charged, in the private sector, for a particular service [7].

The results of this review also suggest a link between the time spent by the practitioner and the costs of the services provided. Whilst, often this is expressed in terms of session duration, it may also be reflected in the setting where service provision occurs; for example, outreach physiotherapy services.

There was little information available in the literature on the cost of domestic services including home cleaning, garden maintenance or handyman activities. Contact with service providers indicated that the cost is highly dependent on the time of service provision. Whilst many were prepared to offer a range of the costs and averages of the cost of service have been provided, most stipulated that they would prefer to give a quote estimate when they actually value the work required in the job.

The differences in the cost of services between countries may be attributed to the societal value attributed to particular disciplines and the historical development of the health care system in each country. There is some similarity between the United Kingdom and Australia in the costs of some of the allied health services but the contribution of disciplines has evolved and developed to varying extents in the different communities, with a slightly different focus in terms of particular discipline intervention. An example of this is in the United Kingdom, which tends to highly value the cost of social work services in comparison to the United States, which places a significantly lower hourly rate [11,32,54].

The following limitations have to be considered for this review. Our aim was to specifically synthesise information on costs of allied health and community services in the Australian context, so necessarily, our review has focussed on Australia. For many of the services in the US, Canada, countries of the European Union and New Zealand only peer-reviewed literature sources have been included, whereas for Australia a more comprehensive search has been conducted to include Government reports and established scheduled fee documents.

It should be acknowledged that some smaller studies relied on reported cost estimates from a limited group of service providers in their local setting. This has an impact on the validity of the cost estimates. In addition, where the costs reported were specific to a particular environmental context, there is impact on the ability to generalise the information gathered.

The comprehensiveness of the economic evaluations also varied. This it was felt did not markedly impact on the accuracy of the unit costs reported, as many studies referred to pre-established unit costs of services that were derived from alternate sources, external to the actual study.

Further research is recommended to explore the difference in the fees charged across different regions in Australia and to determine the impact of setting and workforce supply issues on the cost structure of services.

## Conclusion

Economic evaluations require consideration of the costs of total health service use. Accurate estimation of the range of allied health and community services is essential in determining the cost-effectiveness of intervention and prevention programmes. Utilisation of Government established fee schedules as a broad indicator of the cost of services may lead to underestimation of the real costs of services and therefore to inaccuracies in economic evaluation.

## Competing interests

The authors declare that they have no competing interests.

## Authors’ contribution

IF carried out the database search, screened the titles and abstracts, the full text articles and drafted the manuscript. MF Assisted with the database search activities, in the screening of the sample of full text articles and in the drafting of the manuscript. CS Conceived of the study and participated in its design and coordination and helped to draft the manuscript. KH Participated in the design of the study provided technical expertise and helped to draft the manuscript. All authors read and approved the final manuscript.

## Pre-publication history

The pre-publication history for this paper can be accessed here:

http://www.biomedcentral.com/1472-6963/13/69/prepub
